# Supplementary figures and images for: Limited and Strain-Specific Transcriptional and Growth Responses to Acquisition of a Multidrug Resistance Plasmid in Genetically Diverse Escherichia coli Lineages
Source: mSystems. 2021 Apr 27;6(2):e00083-21. doi: 10.1128/mSystems.00083-21 (PMC8092126; doi:10.1128/mSystems.00083-21)

A

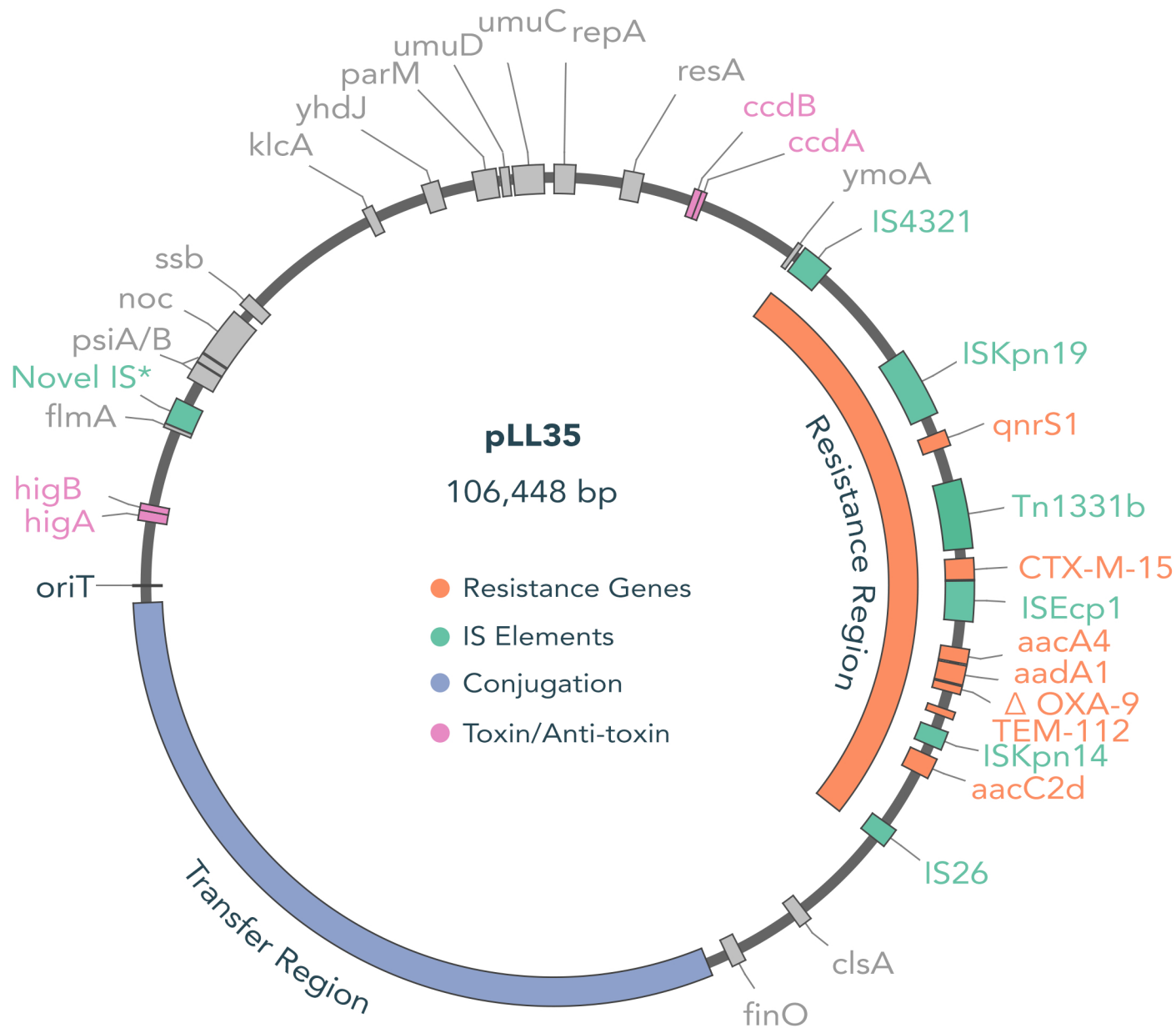

B

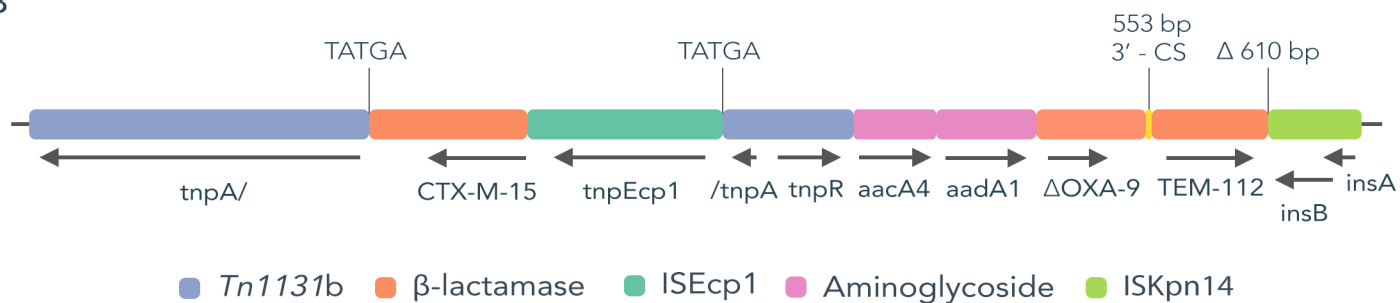

Supplement: FIG S1 [file mSystems.00083-21-sf001.pdf]

log(10) conjugation rate

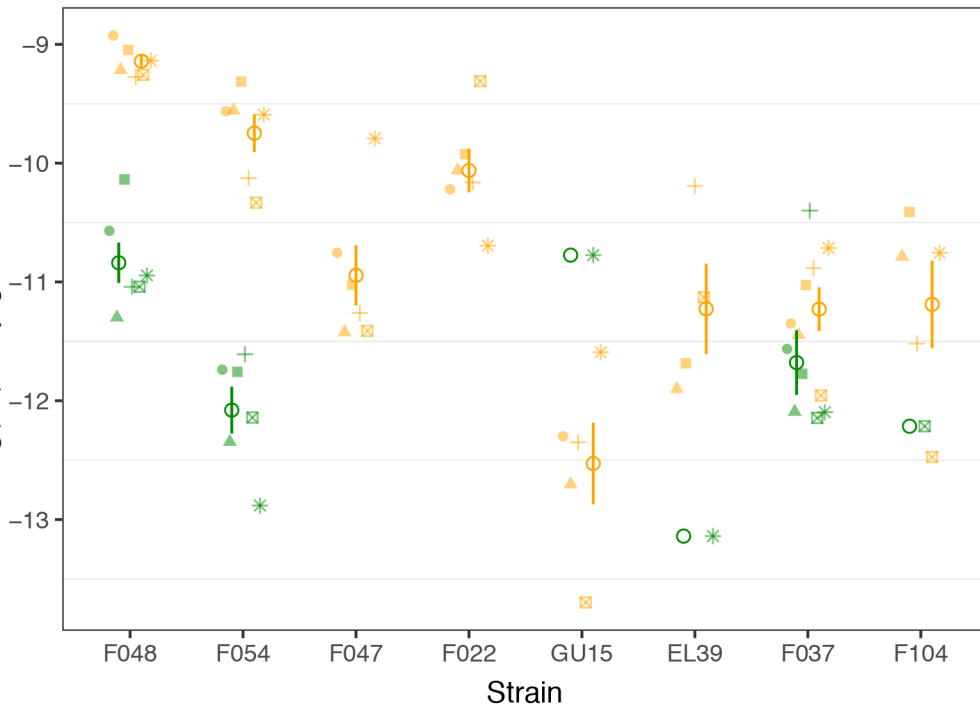

Replicate

- a
- b
- c
- d
- e
- f

Incubation method

- shaking
- static

Supplement: FIG S2 [file mSystems.00083-21-sf002.pdf]

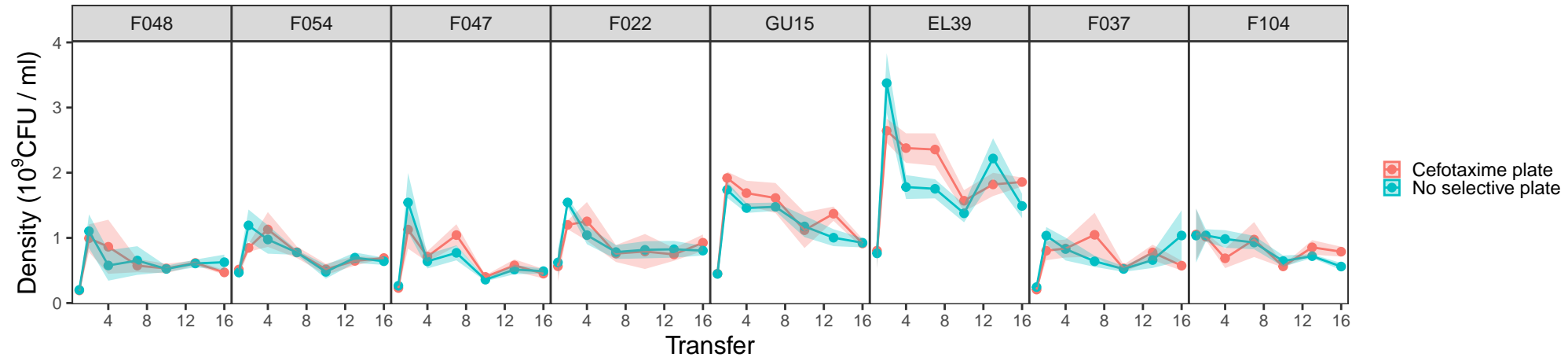

Supplement: FIG S3 [file mSystems.00083-21-sf003.pdf]

A

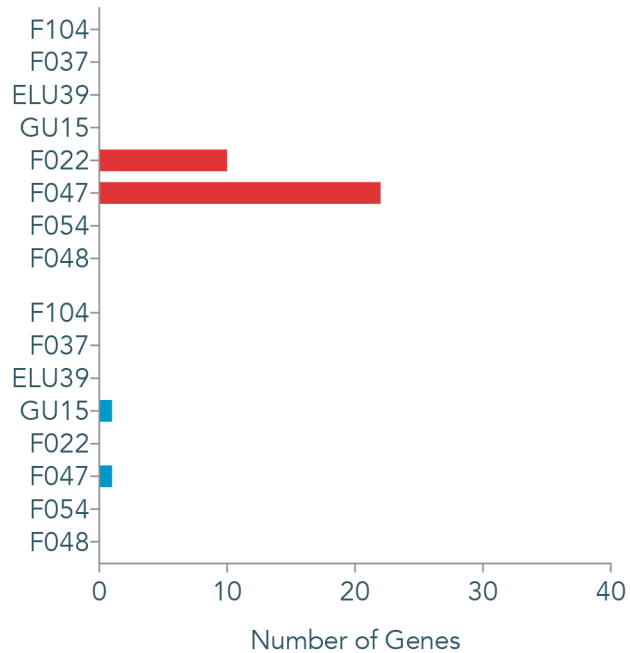

B

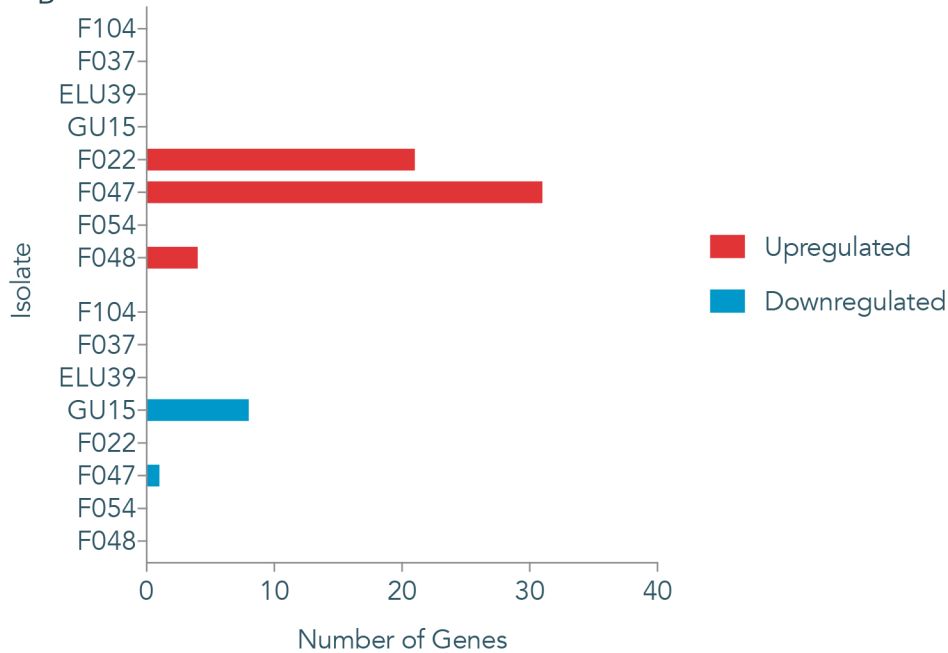

Supplement: FIG S4 [file mSystems.00083-21-sf004.pdf]

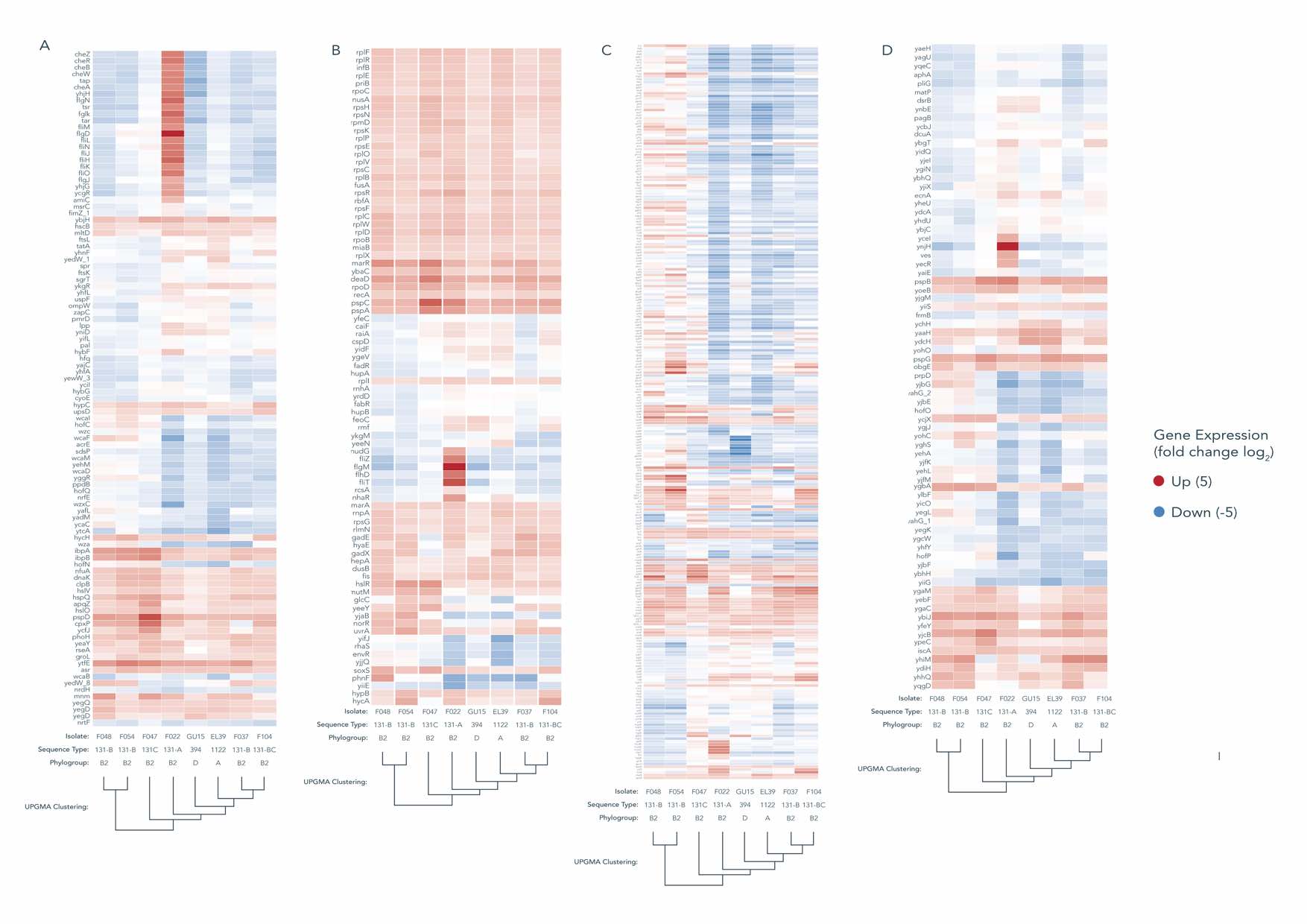

Supplement: FIG S5 [file mSystems.00083-21-sf005.jpg]

A

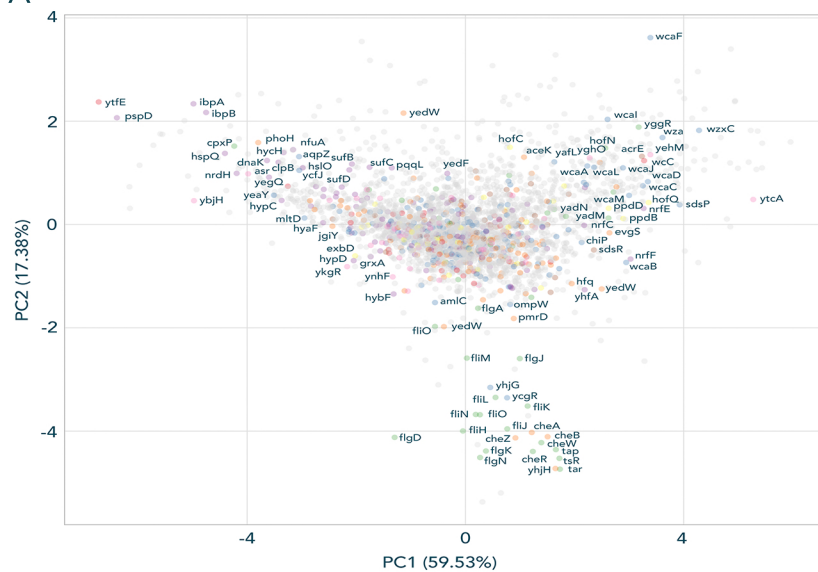

B

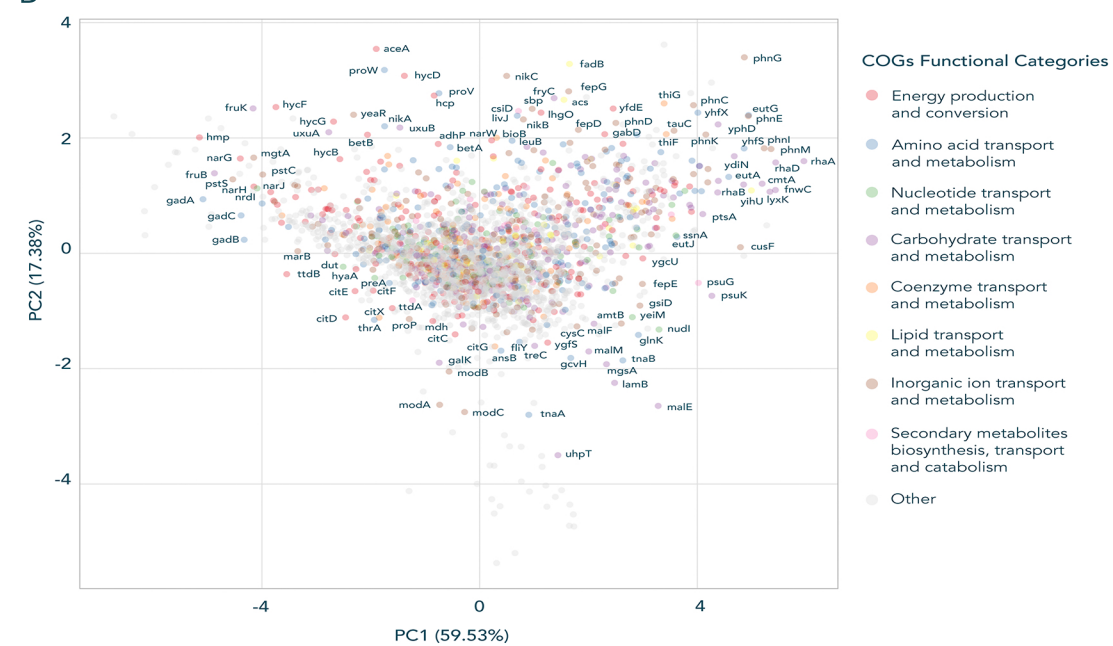

C

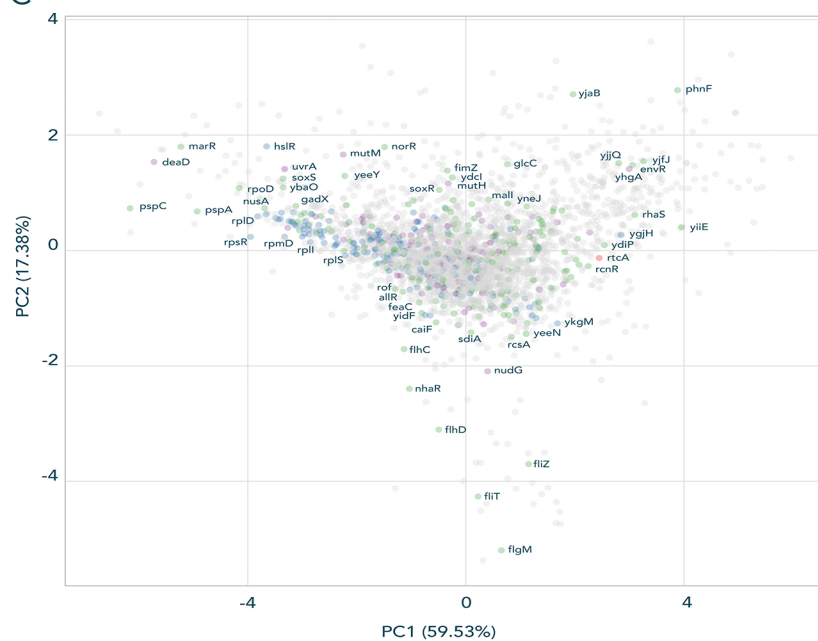

D

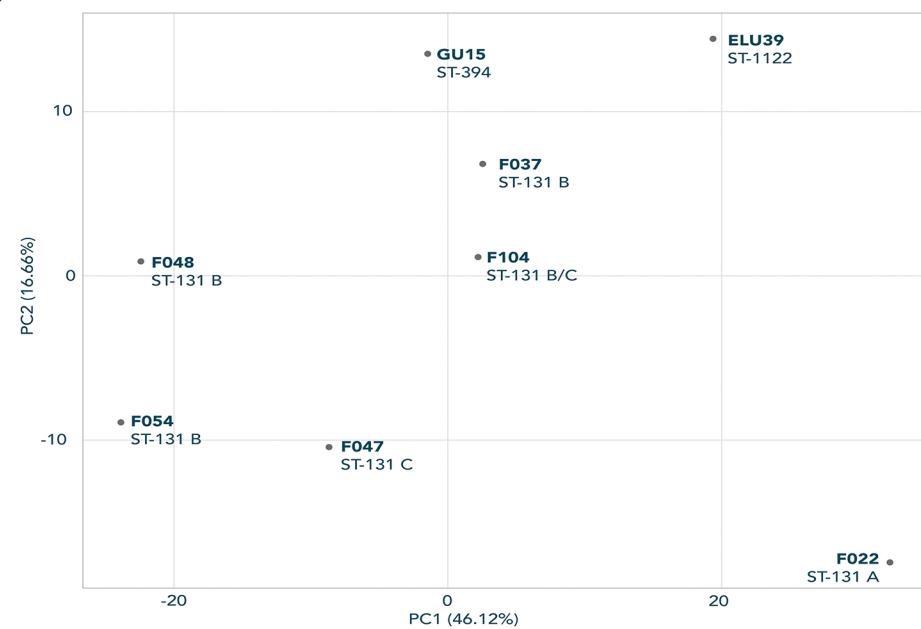

Supplement: FIG S6 [file mSystems.00083-21-sf006.pdf]

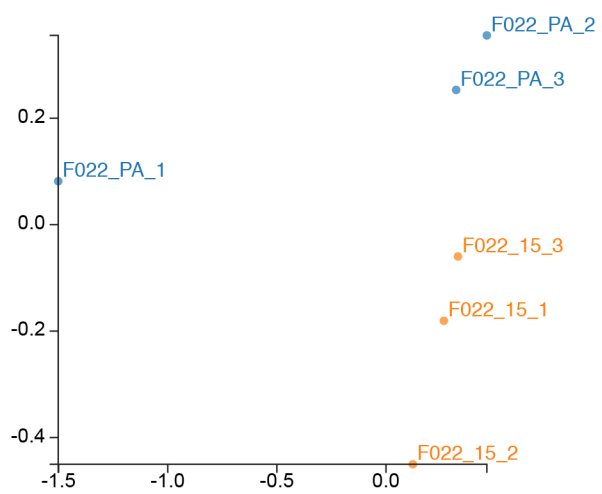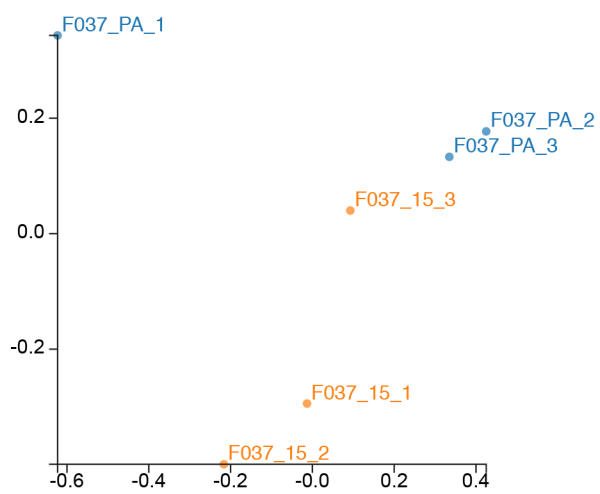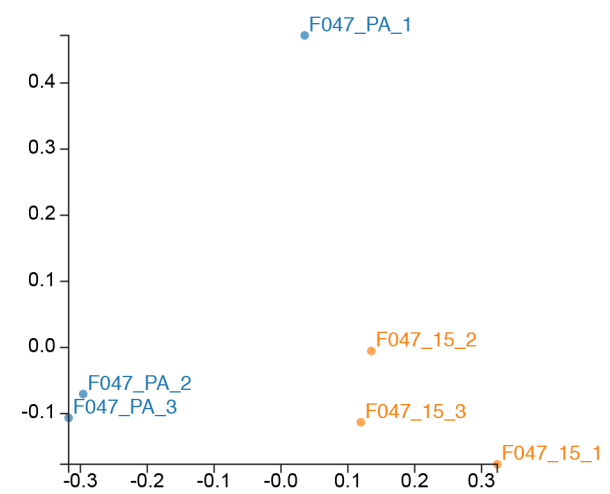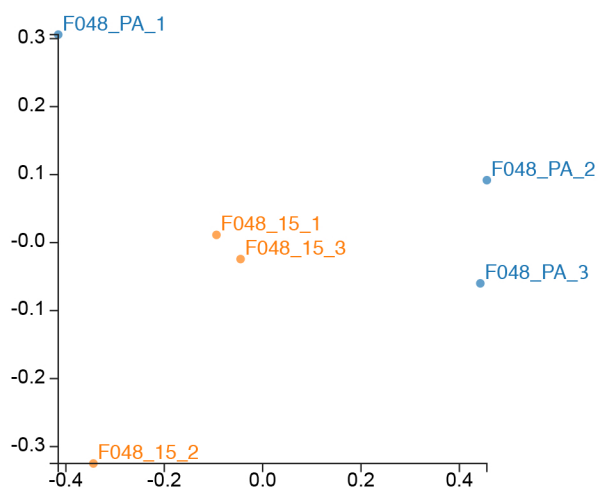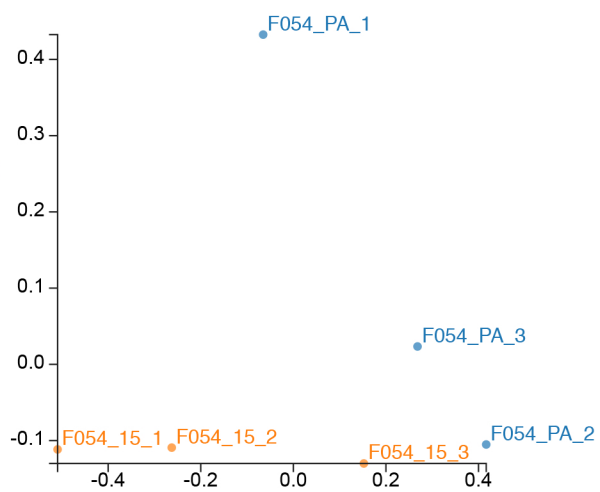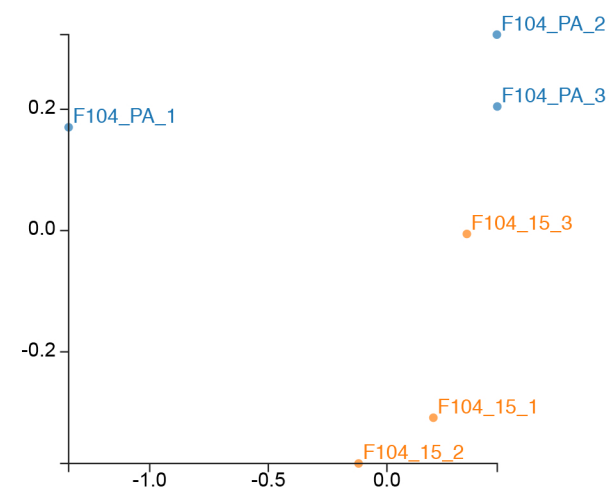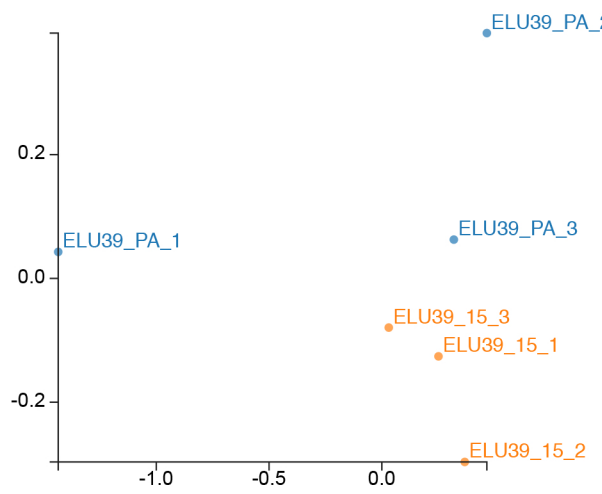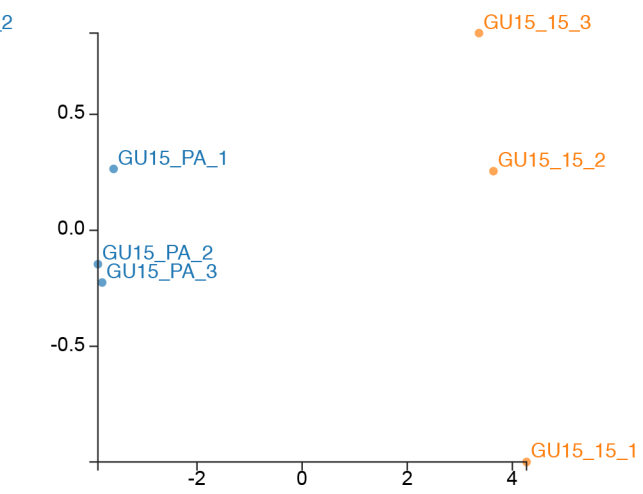

Supplement: FIG S7 [file mSystems.00083-21-sf007.pdf]
